# Inadequate Cerebrospinal Fluid Concentrations of Available Salvage Agents Further Impedes the Optimal Treatment of Multidrug-Resistant *Enterococcus faecium* Meningitis and Bacteremia

**DOI:** 10.3390/idr13030076

**Published:** 2021-09-08

**Authors:** Eric Wenzler, Alina Adeel, Tiffany Wu, Michele Jurkovic, Jeremy Walder, Emily Ramasra, Maureen Campion, Jan Cerny, Nicole M. Theodoropoulos

**Affiliations:** 1 College of Pharmacy, University of Illinois at Chicago, Chicago, IL 60612, USA; twu@lhs.org (T.W.); mjurko2@uic.edu (M.J.); 2 UMass Memorial Medical Center, Division of Infectious Diseases & Immunology, UMass Medical School, Worcester, MA 01605, USA; alina.adeel@umassmemorial.org (A.A.); emramasra@daykimball.org (E.R.); Nicole.Theodoropoulos@umassmemorial.org (N.M.T.); 3 UMass Memorial Medical Center, Department of Internal Medicine, UMass Medical School, Worcester, MA 01605, USA; jeremy.walder@umassmemorial.org; 4 UMass Memorial Medical Center, Department of Pharmacy, Worcester, MA 01605, USA; mcampion@tuftsmedicalcenter.org; 5 UMass Memorial Medical Center, Division of Hematology-Oncology, UMass Medical School, Worcester, MA 01605, USA; jan.cerny@umassmemorial.org

**Keywords:** *Enterococcus faecium*, VRE, meningitis, central nervous system, cerebrospinal fluid, pharmacokinetics, time-kill analysis, case report

## Abstract

Background: *Vancomycin-resistant Enterococcus faecium* (VRE) in particular has evolved as an important cause of hospital acquired infection, especially in immunocompromised hosts. Methods: We present a complex case of a patient with relapsed acute myeloid leukemia who underwent allogenic hematopoietic stem cell transplantation complicated by persistent VRE bacteremia and meningitis. To optimize therapy, various blood and cerebrospinal fluid (CSF) samples were sent to a research laboratory for extensive susceptibility testing, pharmacokinetic analyses, and time-kill experiments. Results: In vitro testing revealed resistance to all first-line treatment options and CSF sampling demonstrated sub-optimal central nervous system concentrations achieved by each antimicrobial agent administered in relation to their respective MIC value. Time-kill analyses at observed CSF concentrations confirmed the lack of bactericidal activity despite use of a four-drug combination regimen. Conclusions: This work is the first to report CSF concentrations of oritavancin and tedizolid in humans and adds to the limited data regarding in vitro susceptibility of new antimicrobial agents such as eravacycline, omadacycline, and lefamulin against VRE. Our study provides new insights into various aspects of treatment of extensively drug-resistant *Enterococcus faecium* meningitis and bacteremia and supports the continued pursuit of precision medicine for these challenging cases.

## 1. Introduction

Although once considered an innocuous commensal organism, serious infections due to *Enterococcus* spp. are now well-described and associated with significant morbidity, mortality, and excess healthcare costs [1]. The difficulty in treating these infections can be attributed in large part to the fact that 30% of all healthcare-associated enterococci and more than 70% of *E. faecium* are vancomycin-resistant (VREf), severely limiting the number of safe and effective treatment options [2]. In addition, their proclivity for vulnerable hosts, predilection for immune-privileged body sites, and complex genotypic–phenotypic relationships have caused them to emerge as one of the most formidable pathogens of the current post-antibiotic era [3]. Furthermore, the emergence of resistance to current first-line treatment agents such as daptomycin and linezolid along with the paucity of high-quality pre-clinical or clinical data supporting alternative treatment regimens has forced clinicians to rely on data from in vitro models, retrospective studies, and case reports when faced with treating extensively resistant VREf infections, particularly in immunocompromised patients [4]. 

These difficulties are further augmented when attempting to treat VREf in less pervious body sites, such as the central nervous system (CNS) [5]. Enterococci are responsible for just 0.3–4% of reported bacterial meningitis cases, with VREf representing exceptionally few of these infections [6]. Notwithstanding pathogen-specific factors, the ability to reach and maintain therapeutic drug concentrations in the CNS is extremely problematic for most antimicrobial agents, including many of those active against VREf. This unfortunate combination of antimicrobial resistance, lack of clinical data and clinician experience, and the dearth of available effective treatment options presents a unique opportunity for collaboration between clinicians and researchers. These collaborations have been employed successfully, especially recently, against a variety of difficult-to-treat pathogens in complex clinical scenarios, highlighting the need for these types of partnerships to optimize therapy and achieve precision medicine [4,7,8,9,10]. Herein, we present a case of an immunocompromised stem cell transplant patient with persistent, extremely drug resistant VREf bacteremia and meningitis treated in collaboration between clinicians and scientists via the use of unique therapeutic drug monitoring and in vitro pharmacokinetic/pharmacodynamic (PK/PD) analyses. 

### Case Presentation

The patient was a 58-year-old man with relapsed acute myeloid leukemia (AML) who achieved remission with salvage chemotherapy and was admitted for a planned haplo-identical stem cell transplant (SCT). In addition to the AML, his previous medical history was significant for subdural hematoma status post craniectomy 9 months prior to this admission and numerous episodes of fever in the setting of neutropenia with associated bacteremia including previous VREf. The first episode of VREf bacteremia occurred 2.5 months prior to the current admission and was susceptible dose-dependent (MIC = 4 mg/L by Etest) to daptomycin and was therefore treated with daptomycin at a dose of 8 mg/kg every 24 h. Blood cultures initially cleared but bacteremia with a daptomycin-resistant VREf (MIC = 32 mg/L by broth microdilution), returned with fevers after 11 days of daptomycin therapy. The isolate remained susceptible to linezolid (MIC = 2 mg/L) and therapy was therefore changed to linezolid 600 mg every 12 h, wherein he successfully completed 29 days of therapy. 

**Figure 1:**
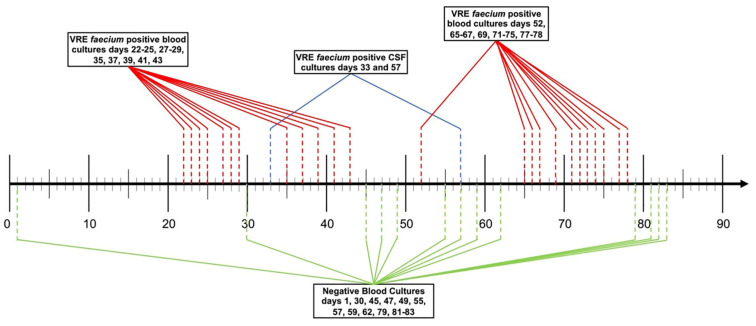
Timeline of VRE culture results from blood and CSF throughout the hospital admission.

**Figure 2:**
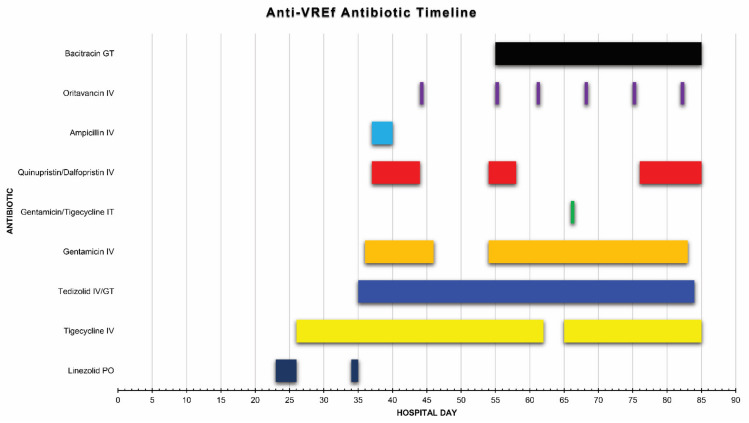
Timeline of antimicrobial regimens administered for the treatment of VREf bacteremia and meningitis throughout the hospital admission. GT, gastrostomy tube; IV, intravenous; IT, intrathecal; PO, per oral.

On hospital day 8 of the current admission, after successful myeloablation with thiotepa/fludarabine/melphalan and post-transplant cyclosphosphamide, he received an allogenic peripheral blood SCT without complication and was started on tacrolimus and sirolimus for graft versus host disease prophylaxis. He remained neutropenic for 20 days but achieved neutrophil engraftment on day 21 post-SCT and remained over 1.0 × 10^3^ cells/uL throughout his hospitalization. On hospital day 22, he developed a fever, blood cultures grew VREf (Figure 1), and he was prescribed linezolid empirically by the transplant infectious diseases team based on prior susceptibilities (Figure 2). Ensuing local laboratory susceptibility testing of this isolate revealed resistance to daptomycin and linezolid but susceptibility to tigecycline. As such, linezolid was switched to tigecycline 100 mg every 12 h as monotherapy while a thorough source investigation was initiated. Central venous catheters were removed, a transthoracic echocardiogram displayed no obvious evidence of valvular dysfunction or vegetations, and a computed tomography (CT) scan of the abdomen and pelvis showed only mild bladder wall thickening. On day 8 of this bacteremia (hospital day 29), he developed acute onset aphasia and a CT of the head showed a new acute on chronic right-sided subdural hematoma resulting in obtundation and status epilepticus. A lumbar puncture (LP) was performed on hospital day 33 and demonstrated pleocytosis (total white blood cell count 480 cells/μL with a neutrophilic predominance of 82%, CSF/blood glucose ratio of 0.16, protein of 120 mg/dL) and a Gram stain containing Gram-positive cocci that subsequently grew VREf on culture along with a blood culture from day 35 (Figure 1). 

Given the persistent bacteremia and new CNS involvement, the day 33 cerebrospinal fluid (CSF) and day 35 blood VREf isolates were sent to the research laboratory for more extensive susceptibility testing (Table 1). On the basis of these results, on hospital day 37, tedizolid 200 mg IV every 12 h, quinupristin-dalfopristin 750 mg IV every 8 h, and gentamicin 5 mg/kg IV were added to tigecycline (IV ampicillin was given briefly for 3 days and discontinued). Blood cultures remained positive for 6 more days on this quadruple combination until he was administered a dose of oritavancin 1200 mg IV on day 44, after which blood cultures cleared the next day and remained negative through to day 52. Quinupristin-dalfopristin and gentamicin were then discontinued, and further source investigation was attempted with an MRI of the cervical, thoracic, and lumbar spine; brain; right shoulder; and abdomen, which were all negative for infectious processes. An Indium-111-tagged white blood cell (WBC) scan on hospital day 49 demonstrated no uptake in the abdomen or pelvis but increased uptake in the lungs and left posterior parietal and occipital regions of the brain. A repeat transthoracic echocardiogram was also performed with good visualization of the valves that did not show any evidence of endocarditis.

On hospital day 52, the patient developed new fever and repeat blood cultures again grew VREf. A repeat LP on day 57 showed improved pleocytosis (WBC 13 cells/μL with a lymphocytic predominance of 89%, CSF/blood glucose ratio of 0.4, and undetectable protein), although CSF cultures again grew VREf (Figure 1). This isolate was also evaluated in the research laboratory and showed similar susceptibilities towards the agents tested against the day 35 isolate (Table 1). Given prior microbiological success, quinupristin-dalfopristin and gentamicin were restarted on hospital day 55 along with weekly oritavancin. Gastrointestinal decontamination via oral administration of bacitracin 25,000 units every 6 h was also attempted (Figure 2). Blood cultures cleared for 10 days on a combination of tigecycline, tedizolid, gentamicin, quinupristin-dalfopristin, oritavancin, and oral bacitracin, although there was no substantial improvement in his neurological status. After blood cultures returned positive again on day 65 (Figure 1), a dose of intrathecal tigecycline 10 mg and intrathecal gentamicin 10 mg were administered on day 66 (Figure 2). Bacteremia persisted for 12 days, but eventually cultures from days 79–83 were negative for VREf (Figure 1). On hospital day 83, after more than 200 combined days of anti-VREf therapy, he developed septic shock requiring multiple vasopressors and blood and sputum cultures grew multi-drug resistant *Pseudomonas aeruginosa*. Meropenem was immediately initiated but the patient quickly decompensated and died on hospital day 84. An autopsy revealed infection with Gram-negative bacilli in his lungs, gastrointestinal tract, and genitourinary tract but no obvious source of persistent VREf infection.

## 2. Materials and Methods

### 2.1. Bacteria and Susceptibility Testing

Initial organism identification and susceptibility determination were performed via MALDI-TOF MS (Bruker Daltonics, Billerica, MA, USA) and MicroScan Walkaway (Beckman Coulter, Brea, CA), respectively, in the clinical microbiology laboratory. Pure colonies of the VREf isolates cultured from the CSF on hospital days 33 and 57 and from blood on days 35 and 52 were sub-cultured to BD BBL chocolate II agar slants (Becton, Dickinson and Company, Sparks, MD, USA) and shipped immediately overnight to the research laboratory where all subsequent testing was performed. Identification and resistance marker detection was confirmed via the Verigene Gram-positive blood culture assay (Luminex, Northbrook, IL, USA). 

Research use only (RUO) MTS strips for lefamulin, omadacycline, quinupristin-dalfopristin, eravacycline (Liofilchem, Roseto degli Abruzzi, Italy); in vitro diagnostic (IVD) Etest strips for fosfomycin (bioMerieux, Marcy-l’Etoile, France); GPALL3F (containing ceftaroline, chloramphenicol, clindamycin, linezolid, penicillin, moxifloxacin, rifampin, tetracycline, tigecycline, gentamicin, erythromycin, ceftriaxone, vancomycin, levofloxacin, oxacillin, ampicillin, telavancin, nitrofurantoin, ciprofloxacin, daptomycin, trimethoprim-sulfamethoxazole, streptomycin); and FDANDPF (containing ceftaroline, clindamycin, dalbavancin, erythromycin, linezolid, oritavancin, oxacillin, tedizolid, telavancin, vancomycin) Sensititre AST plates (ThermoFisher Scientific, Waltham, MA, USA) were utilized according to manufacturer’s instructions. Minimum inhibitory concentrations were determined in triplicate according to CLSI guidelines [11]. Modal MIC values are reported. 

### 2.2. Pharmacokinetic Samples and Analyses

Throughout the duration of systemic gentamicin, serum trough concentrations were measured as part of routine therapeutic drug monitoring. Gentamicin concentrations in plasma were measured via commercially available, validated immunoassay kits (Abbott Laboratories) with a lower limit of quantitation of 0.5 mg/L. From the CSF sample obtained on hospital day 57 as part of standard medical care, two excess CSF tubes were scavenged for the measurement of gentamicin, oritavancin, tigecycline, tedizolid, quinupristin, and dalfopristin concentrations. CSF samples were immediately frozen at −80 °C and shipped overnight on dry ice. Concentrations of each agent in the CSF were quantified via liquid chromatography–tandem mass spectrometry by Keystone Bioanalytical, Inc. (North Wales, PA, USA) with a lower limit of quantitation of 0.001 mg/L. All analytical runs met pre-specified acceptance criteria for standard curve and quality control samples with a coefficient of variation ≤15% and absolute mean bias within 15%. Results are presented as the mean (±SD) of the two measured CSF concentrations. Additionally, estimated corresponding unbound plasma concentrations at the time of CSF collection were extrapolated for each agent from published pharmacokinetic data in order to establish an estimate of the degree of CSF/plasma penetration. This % CSF/plasma penetration was then utilized to predict the maximum post-dose CSF concentration on the basis of the peak free plasma concentration (*f*C_max_) and the time of *f*C_max_ (T_max_) of each agent. Finally, these predictions were evaluated against published data, all of which were restricted to animal studies except for tigecycline [12]. As the bioanalytical analysis of the CSF was not completed prior to the patient expiring, these values and associated time-kill analysis results were not available to the clinical team to inform treatment. 

### 2.3. Time-Kill Experiments

Time-kill experiments were performed in triplicate on the same day as previously described [4] against the VREf isolate cultured from the CSF on hospital day 33. Analytical grade gentamicin, oritavancin, and tigecycline (Sigma-Aldrich, St. Louis, MO, USA), along with tedizolid, dalfopristin, and quinupristin (pristinamycin-IA) (MedChemExpress, Monmouth Junction, NJ, USA) were obtained commercially. Non-tissue culture-treated plates were used, and 0.002% polysorbate-80 was added to all oritavancin assays to prevent any loss of drug potency [13,14]. Antimicrobial agents selected for time-kill analyses alone and in combination were based on the in vitro susceptibility-guided regimen administered to the patient in response to the VREf-positive CSF and blood cultures on day 33 and 35, respectively. The first set of experiments tested each agent alone and in combination at the mean observed CSF concentration obtained from the day 57 lumbar puncture. Next, the 5 drugs were tested in combination at their respective extrapolated maximal CSF concentration as described. 

## 3. Results

### 3.1. Susceptibility

The Verigene platform confirmed the identification of E. faecium and presence of vanA in all four isolates. Phenotypic susceptibilities for each VREf isolate against the tested antimicrobial agents are displayed in Table 1. Overall, the four VREf isolates submitted to the research laboratory were tested against 26 commercially available antibiotics, 23 of which have published clinical breakpoints from the U.S. Food and Drug Administration, Clinical and Laboratory Standards Institute, or the European Committee on Antimicrobial Susceptibility Testing against E. faecalis and/or E. faecium. All four isolates demonstrated high level resistance to both the recommended first-line treatment options daptomycin and linezolid. Other agents lacking VREf-specific clinical breakpoints but that typically maintain reliable activity according to vancomycin-susceptible E. faecalis criteria, including oritavancin and tedizolid, also demonstrated non-susceptible MICs. Tigecycline was the only agent which retained susceptibility against all four VREf. Fosfomycin also demonstrated in vitro susceptibility, although currently available interpretive criteria are based only on the treatment of uncomplicated cystitis due to E. faecalis using the oral tromethamine salt formulation and therefore are not applicable to bloodstream infections. Eravacycline displayed activity against both VREf isolates collected on day 57 but was not tested against the previous set of isolates. Notably, the two sets of corresponding blood and CSF cultures obtained approximately 2.5 weeks apart during this hospitalization demonstrated virtually identical susceptibility profiles despite significant antimicrobial exposure during this time. The only agent for which the MIC changed by >1 log_2_ dilution was quinupristin-dalfopristin, which decreased from 2 mg/L (intermediate) to 0.5 mg/L (susceptible). 

### 3.2. Pharmacokinetics and CSF Penetration

Table 2 displays the mean (±SD) measured CSF concentration of each of the five agents (six compounds) tested in relation to the last dose prior to CSF collection, the estimated corresponding unbound plasma concentration, predicted % CSF/plasma penetration, and the projected maximum CSF concentration. All drugs had been administered systemically for ≥20 days prior to CSF collection and were therefore assumed to be at steady-state plasma conditions. As CSF fluid analysis from day 57 demonstrated an undetectable protein concentration (<1 g/dL), protein binding within the CSF was assumed to be zero for the purposes of estimating % penetration. 

Over the course of 40 days of systemic gentamicin therapy at a dose of 400 mg every 48 h, 18 serum trough concentrations were obtained and all but two were above the lower limit of quantitation. Excluding these two values, the mean (±SD) plasma trough concentration was 0.89 ± 0.5 mg/L. Even with more than 72 h elapsing between the time of the last dose and CSF collection, gentamicin displayed the highest mean CSF concentration of any drug by more than twofold at 0.410 ± 0.05 mg/L, despite undetectable plasma concentrations as evidenced by a trough concentration collected just 8 h prior to CSF collection which was below the lower limit of quantitation. 

Almost exactly 2 days had elapsed between the time of the patient’s last oritavancin dose and the time of CSF collection. The average (±SD) CSF concentration at this time was 0.013 ± 0.005 mg/L. On the basis of the fCmax after a 1200 mg dose of 20.7 mg/L and a half-life of 245 h, the estimated unbound plasma concentration at 48 h post-dose would be approximately 18.8 mg/L. This would result in a 48 h CSF/plasma ratio for this patient of 0.07%, leading to a projected maximal CSF concentration of 0.014 mg/L at plasma T_max_. 

The last doses of tedizolid and tigecycline were administered approximately 8.5 h prior to CSF collection and both demonstrated similar mean (±SD) CSF concentrations at 0.204 ± 0.006 mg/L and 0.172 ± 0.002 mg/L, respectively. Given the reported IV tedizolid steady-state plasma fC_max_ of 0.6 mg/L and 12 h half-life [15], the unbound plasma concentration after 8.5 h would be expected to be ≈0.4 mg/L, leading to a CSF/plasma penetration ratio of approximately 50% and therefore an expected maximum CSF concentration of roughly 0.3 mg/L at T_max_. Although this 50% ratio is notably higher than the 2.16% average observed in uninfected rats [16] and no human data are available, it would be consistent with the CSF penetration reported for linezolid among critically ill patients of 66–85%, especially considering its much lower protein binding of 30% (compared to 80% for tedizolid) [17,18,19,20]. Several published reports exist detailing the CNS penetration of tigecycline, the largest of which evaluated six patients and demonstrated that at 1.5 h and 24 h after a 100 mg dose, the CSF concentrations were 0.015 and 0.025 mg/L, respectively, leading to an average CSF/plasma ratio of 11% [21]. In a separate report of a patient with cerebritis due to Acinetobacter, penetration ratios ranged from 7–36% [22]. Considering the plasma fCmax of 0.3 mg/L after a 100 mg dose and half-life of ≈30 h, the free plasma concentration at 8.5 h would be approximately 0.26 mg/L, making our CSF/plasma ratio 65.5% and corresponding CSF maximum concentration approximately 0.197 mg/L. 

Finally, despite being administered just 2 h prior to CSF collection, concentrations of both quinupristin and dalfopristin were undetectable in all four samples analyzed. This is consistent with published rabbit pneumococcal meningitis data [23] and supports the intrathecal or intraventricular use of this agent over systemic administration when appropriate [24]. 

### 3.3. Time-Kill Analyses

**Figure 3:**
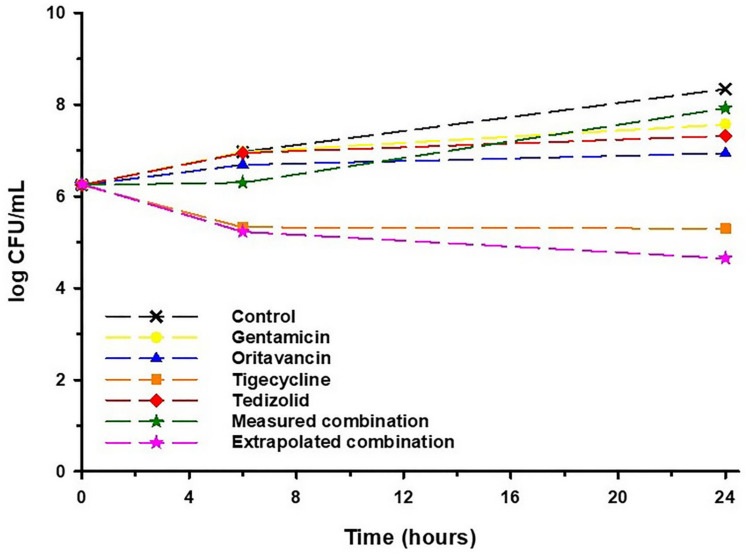
Mean log_10_ cfu/mL versus time profile for each individual drug alone and in combination at their respective measured CSF concentration and extrapolated maximal CSF concentration against the VREf isolate cultured from CSF on hospital day 33. Curves represent average concentrations for triplicate experiments.

Time-kill analyses were performed on the VREf isolate obtained from the CSF on hospital day 33 (Table 1) against each of the four agents being used for treatment at that time both alone and in combination. Quinupristin/dalfopristin was not included as CSF concentrations were undetectable. In attempt to simulate the antibacterial activity occurring in vivo, we tested each drug alone and in combination at the measured mean CSF concentration and again in combination at the projected maximum CSF concentration, as detailed in Table 2. Results of time-kill experiments of each agent alone and in combination at their respective mean measured CSF concentration and in combination at their estimated maximum CSF concentrations are displayed in Figure 3. No single agent or combination was able to achieve bactericidal activity. Consistent with its measured CSF concentration/MIC ratio compared to the other agents tested, tigecycline was the only agent capable of reducing the bacterial inoculum (decrease of 0.95 log_10_ colony-forming units (CFU)/mL at 24 h compared to the starting concentration). Oritavancin was the second most active agent, allowing 0.69 log_10_ CFU/mL of growth by 24 h. Treatment with either gentamicin or tedizolid alone resulted in an increase of >1 log_10_ CFU/mL compared to the starting inoculum. When these four agents were combined at their respective measured CSF concentrations, >1.5 log_10_ CFU/mL of bacterial growth was observed over 24 h compared to the starting inoculum. Finally, when combined at the drugs’ respective estimated maximum CSF concentrations, the four-drug combination demonstrated the greatest antibacterial activity, resulting in a 1.62 log_10_ CFU/mL reduction at 24 h (Figure 3). 

## 4. Discussion

Despite the advent of new agents with in vitro activity against VREf, the morbidity and mortality associated with serious infections remains unacceptably high, due in large part to increasing antimicrobial resistance and the dearth of adequate data to inform treatment [28,29]. This is particularly true in the case of CNS infections, where the paucity of available agents becomes even more limited and the mortality is higher due to neuroanatomical obstacles limiting drug penetration and impeding host defense mechanisms. Since achieving therapeutic antibacterial concentrations at the site of infection is critical for optimizing efficacy [30], it is crucial to develop a thorough understanding of the PK and PD properties of these agents to treat an individual patient more precisely. Unfortunately, the lack of an established regulatory pathway and the significant cost and complexity associated with performing controlled clinical trials in patients with CNS infections ensures that any understanding of the PK/PD properties or the safety and efficacy of these agents is likely to be gleaned only from observational reports and case series. Further, guidelines from the Infectious Diseases Society of America on bacterial meningitis are from 2004 and therefore antiquated, while the 2017 guidelines for healthcare-associated ventriculitis and meningitis do not address the treatment of VREf [31,32]. Consequently, the responsibility ultimately falls on the well-educated practitioner to assess all available scientific literature and select the most appropriate therapy for this deadly pathogen–disease combination. These complex clinical scenarios with little useful existing data lend themselves well to facilitating collaborations between clinicians and scientists whose efforts can synergize to improve the care of current and future patients. 

Herein, we describe the case of an immunocompromised patient with persistent, extensively drug-resistant VREf bacteremia and meningitis who was unable to sustain a clinical or microbiological cure despite combination therapy with up to five agents guided by extensive in vitro susceptibility testing. This lack of efficacy is likely explained in part by the suboptimal CNS concentrations achieved by each agent (Table 2), especially in relation to their respective MIC values against the VREf isolates obtained from this patient (Table 1). This was confirmed by our time-kill analyses, demonstrating no bactericidal activity even at the projected maximal CSF concentrations (Figure 3). This is further illustrated by the lack of VREf infection found on autopsy, suggesting that antimicrobial therapy may have been effective at eradicating the pathogen from extravascular tissue where drug concentrations were likely sufficiently high, but not in the CSF. Importantly, to our knowledge this is the first study to report CSF concentrations of oritavancin and tedizolid in humans and one of exceedingly few to measure CSF concentrations of any anti-VREf agent in a patient without an indwelling device such as an extraventricular drain or ventriculoperitoneal shunt [5,6,33]. Our work also adds to the currently limited data regarding the in vitro susceptibility of new agents against resistant strains of VREf, specifically eravacycline, omadacycline, and lefamulin [34,35,36,37,38]. Currently, no in vitro or in vivo PK/PD data are available to guide their use against VREf, and only eravacycline has data regarding the CNS penetration in rabbits which was virtually zero [39]. Finally, the current study expands on our groups’ previous work by evaluating additional combination therapies against daptomycin and linezolid-resistant VREf in time-kill analyses and confirming the sustainability and utility of this clinician–scientist collaboration [4].

Limitations to this work include the lack of repeated PK measurements, absence of concomitant plasma concentrations to correlate with CNS concentrations, and lack of whole genome sequencing analysis to identify genetic mechanisms of resistance. Moreover, the lack of inflammation present on the day 33 CSF sample likely contributed in part to the low drug concentrations observed in addition to the physiochemical properties of the agents tested such as the large molecular weight and high protein binding of oritavancin. Finally, despite that the results of our PK/PD analysis were not available in real time to assist with this patient’s care, they likely would not have changed their antimicrobial regimen as therapy was already optimized to the extent possible based on our in vitro susceptibility testing. 

## 5. Conclusions

The promise of precision antimicrobial therapy is that a deeper understanding of the PK/PD properties of these agents alone and in combination can guide us towards improved therapies and better patient outcomes. Although the collaboration utilized herein to achieve this goal is not currently universally applicable, it is a step towards improving the quality of care and combating antimicrobial resistance. We hope that these data are beneficial in helping future patients afflicted with this challenging pathogen. 

## Figures and Tables

**Table 1:** Phenotypic susceptibilities and interpretive categorya of VREf isolates to tested antimicrobials ^a^.

Source (Hospital Day of Isolation)	CSF (33)		Blood (35)		CSF (57)		Blood (52)	
Antibiotic	MIC (mg/L)	Interpretive Category	MIC (mg/L)	Interpretive Category	MIC (mg/L)	Interpretive Category	MIC (mg/L)	Interpretive Category
Ampicillin	≥16	R	≥16	R	≥16	R	≥16	R
Ceftaroline	≥128	NC	≥128	NC	≥128	NC	≥128	NC
Ceftriaxone	≥128	NC	≥128	NC	≥128	NC	≥128	NC
Chloramphenicol	≥32	R	≥32	R	≥32	R	≥32	R
Ciprofloxacin	≥4	R	≥4	R	≥4	R	≥4	R
Dalbavancin ^b^	≥4	NS	≥4	NS	≥4	NS	≥4	NS
Daptomycin	≥8	R	≥8	R	≥8	R	≥8	R
Eravacycline ^d^	-	-	-	-	0.12	S	0.12	S
Delafloxacin ^c^	>8	R	>8	R	>8	R	>8	R
Erythromycin	≥8	R	≥8	R	≥8	R	≥8	R
Fosfomycin ^b^	64	S	64	S	64	S	64	S
Gentamicin synergy	≤500	S	≤500	S	≤500	S	≤500	S
Lefamulin	1	NC	1	NC	2	NC	2	NC
Levofloxacin	≥8	R	≥8	R	≥8	R	≥8	R
Linezolid	16	R	16	R	16	R	16	R
Moxifloxacin ^c^	≥8	R	≥8	R	≥8	R	≥8	R
Nitrofurantoin	≥128	R	≥128	R	≥128	R	≥128	R
Omadacycline ^c^	1	R	1	R	1	R	1	R
Oritavancin ^b^	0.25	NS	0.25	NS	0.25	NS	0.25	NS
Quinupristin-dalfopristin	2	I	2	I	0.5	S	0.5	S
Rifampin	≥4	R	≥4	R	≥4	R	≥4	R
Tedizolid ^b^	2	NS	2	NS	2	NS	2	NS
Telavancin	≥4	NS	≥4	NS	≥4	NS	≥4	NS
Tetracycline	≥16	R	≥16	R	≥16	R	≥16	R
Tigecycline ^d^	0.25	S	0.25	S	0.25	S	0.25	S
Vancomycin	≥256	R	≥256	R	≥256	R	≥256	R

-, not tested; NC, no CLSI or EUCAST interpretive category; S, susceptible; I, intermediate; R, resistant; NS, non-susceptible. ^a^ According to CLSI M100-S30 unless otherwise specified. ^b^ Interpreted according to CLSI breakpoint for vancomycin-susceptible *E. faecalis.* ^c^ Interpreted according to FDA breakpoints for *E. faecalis.* ^d^ Interpreted according to EUCAST clinical breakpoints v.10.0 for *E. faecium.*

**Table 2:** Mean (±SD) measured CSF concentrations of each agent quantified from the day 57 lumbar puncture along with estimated maximum CSF concentrations and CSF/plasma penetration ratios.

Antimicrobial	Last Dose Administered (mg)	Time between Last Dose and CSF Collection (h:m)	Mean (±SD) Measured CSF Concentration (mg/L)	Estimated Corresponding Unbound Plasma Concentration Range at Time of CSF Collection (mg/L)	Predicted CSF/Plasma Penetration (%)	Projected Maximum CSF Concentration (mg/L)	Published % CSF/Plasma Penetration (REF)
Gentamicin	400	77:30	0.410 ± 0.05	ND	-	5.74	27 [25]
Oritavancin	1200	46:54	0.013 ± 0.005	15.5–25.1	0.07	0.01–0.02	1–5 [26]
Tigecycline	100	8:27	0.172 ± 0.002	0.16–0.46	71.7	0.27–0.78	5–41 [21,22]
Tedizolid	200	8:25	0.204 ± 0.006	0.30–0.49	54.8	0.25–0.41	2.2 [16]
Quinupristin	207	2:00	ND	0.33–0.43	0	0	0 [23,27]
Dalfopristin	483	2:00	ND	1.84–3.04	0	0	0 [23,27]

ND, not detected.

## Data Availability

The data that support the findings of this study are available on request from the corresponding author and are not publicly available due to privacy or ethical restrictions.
